# Functionalization of a Shape-Memory 3D-Printed Clear Aligner Resin with Silanized Chitosan Nanoparticles: Antibacterial, Optical, Polymerization, and Mechanical Evaluation

**DOI:** 10.3390/jfb17070345

**Published:** 2026-07-16

**Authors:** Saya Mustafa Azeez, Anees Mahmood Mudhir

**Affiliations:** 1 Department of Preventive, Orthodontics, and Pediatric Dentistry, College of Dentistry, University of Duhok, Duhok 42001, Iraq; anees.mudhir@uod.ac; 2 Department of Orthodontics, Faculty of Dentistry, Tishk International University, Erbil 44001, Iraq

**Keywords:** orthodontic appliances, dental materials, bacterial adherence, biofilm, tensile strength, flexural strength

## Abstract

Directly 3D-printed clear aligners are increasingly adopted for orthodontic treatment, but lack intrinsic antibacterial properties. This study investigates the integration of silanized chitosan nanoparticles (CSNPs) into 3D-printed clear aligner resin to improve antibacterial activity while preserving optical, polymerization, and mechanical performance. Commercial CSNPs were surface modified with 3-(trimethoxysilyl)propyl methacrylate and characterized using FTIR, UV-Vis, TEM, EDS, XRD, DLS and zeta-potential analysis. The silanized CSNPs were incorporated into TA-28 clear aligner resin at 0.01, 0.05, and 0.1 wt%. Antibacterial activity against Streptococcus mutans (*S. mutans*) and Porphyromonas gingivalis (*P. gingivalis*) was assessed using bacterial adherence and biofilm assay. Transparency, degree of conversion, tensile and flexural properties were evaluated. Successful silanization was confirmed by characterization analyses. The incorporation of silanized CSNPs reduced bacterial adherence and biofilm formation significantly in comparison with the control group (*p* < 0.001). The transparency was maintained at 0.01 and 0.05 wt% but reduced significantly at 0.1 wt% (*p* < 0.001). While all nano-modified groups had a lower DC% than the control group, values remained above 75%. Mechanical performance showed improvements, particularly in stiffness and flexural properties, following nanoparticle incorporation. Among the tested concentrations, the 0.05 wt% CSNPs provided the most favorable overall performance, highlighting their potential for the development of next-generation bioactive clear aligners.

## 1. Introduction

Removable clear orthodontic aligners that are custom-fabricated, transparent, and designed to gradually move the teeth have gained wide popularity over conventional fixed orthodontic therapy as they are more aesthetic and comfortable [1]. Although they provide several benefits, such as removability and better oral hygiene maintenance, they are worn for around 22 h per day, and their wrap-around design on the tooth’s surface can create favorable conditions for biofilm formation and proliferation of cariogenic microorganisms [2]. In addition, the process of additive manufacturing used to produce these appliances may introduce microscopic voids or surface irregularities, which act as niches for adhesion and colonization of bacteria, potentially further compromising oral hygiene [3].

The natural self-cleansing function of saliva can be interfered with by the presence of a clear aligner in the oral cavity and, consequently, raise the susceptibility of enamel demineralization. Clinically, these alterations are commonly manifested as white spot lesions (WSLs) [4]. A previous study found that 1.2% of patients with clear aligners develop WSLs in comparison with 26% of patients with conventional fixed orthodontic therapy, indicating that clear aligner therapy minimizes but does not completely eliminate the risk of enamel demineralization [4].

*Streptococcus mutans* (*S. mutans*) and *Porphyromonas gingivalis* (*P. gingivalis*) are microorganisms that play a particularly important role in biofilm formation on orthodontic appliances. *S. mutans* is a major Gram-positive cariogenic bacterium responsible for WSLs and formation of caries because of its capacity to adhere to the tooth and polymer surface, making extracellular polysaccharides and fermenting carbohydrates into acids [5]. Meanwhile, *P. gingivalis* is a Gram-negative anaerobic oral pathogen that is associated with periodontitis, a chronic inflammatory condition that leads to destruction of the supporting structure of the teeth, involving periodontal ligaments and alveolar bone. If left untreated, it eventually leads to tooth loss [6]. The close adaptation between the clear aligner and the tooth surface, along with reduced salivary flow underneath the aligner, can lead to microbial retention and persistence at the aligner–tooth interface, possibly increasing the risk of cariogenic and periodontal biofilm development [7].

Recent progress in 3D printing and computer-aided design technologies has enabled the fabrication of direct-printed aligners. This approach offers several advantages, including greater manufacturing accuracy and precision, a more streamlined production workflow, and reduced material waste [8]. Among these materials, Tera Harz TA-28 resin (Graphy Inc., Seoul, Republic of Korea) has been recently introduced as an updated version of Graphy’s earlier TC-85 resin, with enhanced material properties. Shape memory is one of its properties, which allows the material to deform and return to its original configuration and maintain stable orthodontic force during treatment [9]. However, the incorporation of nanoparticles to introduce antimicrobial properties into clear aligners remains relatively underexplored.

In biomedical research, nanoparticles have gained considerable attention due to their unique physicochemical properties [10]. In dentistry, they have been widely explored for incorporation into polymeric materials due to their antibacterial properties, remineralization potential and capability for drug delivery [11]. The addition of nanoparticles into dental material can also improve mechanical properties, surface characteristics and wear resistance, enhancing overall bioactivity [12].

Chitosan is a natural polysaccharide acquired through the alkaline deacetylation of chitin, which is found in crustacean shells, insect cuticles, and arthropod exoskeletons. Chitosan and its nanoparticle form have gained attention in diverse fields owing to their biodegradability, biocompatibility, and lack of toxicity [13]. Furthermore, it exhibits intrinsic antimicrobial properties. Its cationic amino groups enable electrostatic interaction with negatively charged bacterial cell walls, which increases the permeability of the membrane and leakage of intracellular components, eventually leading to cell death. Moreover, it may interfere with mRNA transcription and protein formation in microbial cells [13,14,15].

This study aimed to investigate the integration of chitosan nanoparticles (CSNPs) into 3D-printed clear aligner material to develop an appliance with enhanced antimicrobial properties. The CSNPs were surface-modified with silane coupling agent 3-(trimethoxysilyl) propyl methacrylate (TMSPM) to improve their compatibility with the polymer matrix. Characterization tests were performed to confirm successful silanization, followed by evaluation of antibacterial activity and physical properties, such as the transparency, degree of conversion and mechanical properties of the modified 3D-printed clear aligner.

## 2. Materials and Methods

### 2.1. Study Design and Sample Size

This in vitro experimental study evaluated the effect of incorporating silanized CSNPs (Section 2.2) into commercially available 3D-printed clear aligner resin (Tera Harz TA-28, Graphy Inc., Seoul, Republic of Korea), a urethane methacrylate-based shape-memory photopolymer. Based on the MIC/MBC findings (Section 2.5), which demonstrated the antibacterial activity of the silanized CSNPs at relatively low concentrations, three nanoparticle concentrations (0.01, 0.05, and 0.1 wt%) were selected to provide antibacterial activity while minimizing potential adverse effects on the optical, polymerization, and mechanical properties of the resin. Four experimental groups were investigated: Group 1 (control), unmodified TA-28 resin; Group 2, TA-28 resin + 0.01 wt% silanized CSNPs; Group 3, TA-28 resin + 0.05 wt% silanized CSNPs; and Group 4, TA-28 resin + 0.1 wt% silanized CSNPs. Specimens were designed according to the requirements of each test.

Three specimens per subgroup were selected based on previous in vitro microbiological studies [16,17]. The required sample size for mechanical, transparency and degree-of-conversion tests was calculated using the G*Power software (v3.1.9.4, Universität Kiel, Germany). A minimum of nine specimens per subgroup was calculated to be adequate. This estimation was based on an effect size of 0.5, α = 0.05, and power = 80%.

### 2.2. Preparation and Silanization of Chitosan Nanoparticles

A 100 mL ethanol solution (70 vol%) was prepared by mixing ethanol (99.8%) with deionized water, and the pH was adjusted to 4.5 by titrating with 99.9% acetic acid. The silane coupling agent 3-(trimethoxysilyl) propyl methacrylate (TMSPM) (CAS no.: 2530-85-0, Thermo Scientific, Loughborough, UK) was subsequently added to the ethanol solution at 2 wt% relative to the chitosan nanoparticle (CSNP) mass (0.3 mL for 15 g nanoparticles) and magnetically stirred. Commercial CSNPs (80–100 nm; Nanochemazone, Leduc, Alberta, Canada) were subsequently dispersed in the hydrolyzed silane solution, followed by magnetic stirring for 20 min and then probe sonication for 30 min. The suspension was centrifuged (Pro-hospital, Centurion scientific, Chichester, West Sussex, UK) at 6000 rpm for 10 min, the supernatant containing ethanol was discarded, and the collected silanized CSNPs were dried using a rotary evaporator (RE-2000E, Biobase Co., Jinan, China) at 60 °C for 30 min [18].

### 2.3. Physicochemical Characterization of Chitosan Nanoparticles Before and After Silanization

Several characterization methods were used to assess the physicochemical properties of the CSNPs and following surface modification with TMSPM. UV–visible spectroscopy, attenuated total reflectance–Fourier-transform infrared (ATR-FTIR) spectroscopy, X-ray diffraction (XRD), transmission electron microscopy (TEM), and energy-dispersive spectroscopy (EDS) were among the analyses used. Additionally, zeta potential, size distribution, and particle size were measured to evaluate the colloidal properties of the nanoparticles.

UV–visible absorption spectra (190–800 nm) were acquired using a BK-D560 (Biobase Co., Jinan, China). The nanoparticles were dispersed in a 0.1% (*v*/*v*) acetic acid suspension. The chemical structure and surface modification of CSNPs were evaluated by ATR-FTIR spectroscopy (PerkinElmer Inc., Shelton, CT, USA). The spectra were obtained from 4000 to 400 cm^−1^ at a resolution of 4 cm^−1^ using 32 scans per spectrum. The crystalline structure of the nanoparticles was analyzed by XRD (X’Pert pro diffractometer, PANalytical, Netherlands) operating at 40 kV and 30 mA, using Cu Kα radiation (λ = 1.5406 Å) across a 2θ range of 5–80°, with a step size of 0.02°.

Using TEM (Zeiss Leo 912 AB, Oberkochen, Germany), the nanoparticle shape and size distribution were analyzed. To minimize agglomeration, a small quantity of the nanoparticle powder was dissolved in ethanol and sonicated for 10 min prior to imaging. Before TEM examination, a drop of the suspension was put on a copper grid coated with carbon and allowed to dry at ambient temperature. The elemental composition was analyzed using a Bruker (Billerica, MA, USA) system. To reduce the surface charge, the nanoparticles were sputter-coated with a thin layer of platinum and then mounted on aluminum stubs utilizing conductive carbon tape and examined under vacuum conditions.

At 25 °C, measurements of particle size, size distribution, and zeta potential were made (Amerigo, Cordouan Technologies, Pessac, France). The CSNPs and silanized CSNPs were disseminated in 0.1% (*v*/*v*) acetic acid, sonicated to create a homogenous solution, and then diluted in 1 mM KCl electrolyte that had been adjusted to pH 4.8. Dynamic light scattering (DLS) was used to measure particle size and size distribution. The hydrodynamic diameter (Z-average) and polydispersity index (PDI) were computed from the Brownian motion of particles using the cumulants approach. The Smoluchowski model was used to translate electrophoretic mobility measurements into zeta-potential values, which were calculated using laser Doppler electrophoresis.

### 2.4. Bacterial Culture Preparation

*S. mutans* (ATCC 25175) was rehydrated in brain heart infusion broth (Jinan Babio Biotechnology Co., Jinan, China) and incubated for 48 h, and *P. gingivalis* (ATCC 33277) was rehydrated in tryptic soy broth (HiMedia laboratories Pvt. Ltd., Maharashtra, India) supplemented with hemin (Molekula, Newton Aycliffe, Durham, UK) and vitamin K1 (Amriya Pharmaceutical, Alexandria, Egypt) for 5 days at 37 °C. The bacterial suspensions were standardized to OD600 = 0.1 (~1.5 × 10^8^ CFU/mL, equivalent to 0.5 McFarland) before the antibacterial testing [19].

### 2.5. Minimum Inhibitory Concentration (MIC) and Minimum Bactericidal Concentration (MBC)

The antibacterial activity of the CSNPs and silanized CSNPs was assessed against *S. mutans* and *P. gingivalis* using the broth microdilution method. Two-fold serial dilutions from 1 mg/mL to 0.0625 mg/mL were prepared in Mueller–Hinton broth (HiMedia laboratories Pvt. Ltd., Maharashtra, India), supplemented with hemin and vitamin K for *P. gingivalis* [20]. The bacterial suspensions were adjusted to 0.5 McFarland (1.5 × 10^8^ CFU/mL). A positive control (broth + bacteria), a negative control (broth only), and a nanoparticle control (broth + nanoparticles) were included.

*P. gingivalis* was incubated in an anaerobic environment, while *S. mutans* was incubated in an aerobic environment. The optical density was measured at 630 nm using a microplate reader (ELX-800, BioTek, Winooski, VT, USA) before and after incubation for 24 h at 37 °C. The MIC was defined as the concentration at which there was no increase in absorbance when compared with the growth control.

The MBC was evaluated by subculturing aliquots (50 µL) from wells showing no visible growth on a Mueller–Hinton agar plate (HiMedia laboratories Pvt. Ltd., Maharashtra, India), supplemented with hemin and vitamin K for *P. gingivalis*. *S. mutans* was incubated aerobically for 48 h, while *P. gingivalis* was incubated anaerobically for 5 days at 37 °C. The MBC was determined to have the lowest concentration of nanoparticles that showed no detectable bacterial colony growth on the agar (≥99.9% bacterial reduction).

### 2.6. Preparation of Nanocomposite Resin and Fabrication of Samples

Tera Harz TA-28 (Graphy Inc., Seoul, Republic of Korea) is a commercially available shape-memory photocurable resin used for direct 3D printing of clear aligners. According to the manufacturer, TA-28 is an updated formulation of the earlier Tera Harz TC-85 resin, developed to improve its mechanical properties and degree of conversion. The exact chemical formulation is proprietary and has not been disclosed by the manufacturer. However, according to Choi et al. [21], the closely related TC-85 resin contains oligomers GR30860 and GR3060 and uses bis(2,4,6-trimethylbenzoyl)-phenylphosphine oxide (Irgacure 819; BASF SE, Ludwigshafen, Germany) as the photoinitiator. The primary component of TC-85 is an aliphatic urethane acrylate oligomer that polymerizes to a cross-linked network during photopolymerization and is responsible for shape-memory properties.

The silanized CSNPs were incorporated into TA-28 clear aligner resin at concentrations of 0.01, 0.05, and 0.1 wt%. The nanoparticles were gradually added to the clear aligner resin to minimize aggregation and enhance dispersion. A 5 mm probe was used to sonicate the mixture at 70% power with a 5 s on/off pulse cycle (UCD-PO1, Biobase Co., Jinan, China). To avoid overheating, the beaker was placed in an ice bath, and the probe tip was maintained at ~10 mm above the beaker bottom.

Samples were designed (Autodesk 3ds Max) according to each test requirement and exported as STL files. The files were imported into slicing software (v1.5.4.35, UNIZ Dental) and printed with a UNIZ NBEE LCD 3D printer (San Diego, CA, USA) with a layer thickness of 100 µm. All specimens were printed in a horizontal (0°) orientation, with the long axis parallel to the build platform and the tested surface facing upward. This orientation was selected to ensure that the loading during mechanical testing was applied perpendicular to the printing layers, allowing evaluation of interlayer bonding [22]. It also provided a smooth, support-free surface and allowed comparison with previous studies that adopted the same conditions [23]. Post-processing was performed according to the manufacturer’s instructions for TA-28 resin, which does not include solvent washing, since isopropanol rinsing has been reported to alter mechanical properties [24] and optical properties and increase surface roughness [25]. After printing, the residual uncured resin was removed mechanically by centrifugation at 500 rpm for 6 min at room temperature using a Tera Harz Spinner (THSP, Graphy, Seoul, Republic of Korea), followed by post-curing under a nitrogen atmosphere for 20 min using a Tera Harz Cure unit (THC, Graphy, Seoul, Republic of Korea) to minimize oxygen inhibition and maximize polymerization.

### 2.7. Bacterial Adherence Assay

Sterilized disc-shaped specimens (*n* = 3; 10 mm × 2 mm) were placed in a 24-well plate, and each well received *S. mutans* and *P. gingivalis* suspensions (1.5 × 10^8^ CFU/mL) and was incubated at 37 °C aerobically and anaerobically for 24 h to allow initial bacterial attachment, respectively. A positive control (broth + bacteria) and a negative control (broth only) were included. Afterwards, the samples were washed three times with phosphate-buffered saline (PBS) to remove the non-adhered bacteria and then bath-sonicated (60 s) to detach the adherent bacteria in PBS solution [26].

A two-step serial dilution step (1:50 each) was performed for *S. mutans*, and 50 µL of the second dilution was spread onto Mueller–Hinton agar and incubated aerobically at 37 °C for 24 h. A one-step serial dilution (1:50) was performed for *P. gingivalis*, and 50 µL was plated onto Mueller–Hinton agar supplemented with hemin and vitamin K and incubated anaerobically at 37 °C for 5 days. After the incubation period, the visible colonies were counted, and the number of adherent bacteria was calculated as CFU/mL, considering the dilution factor and plated volume.

### 2.8. Biofilm Assay

Suspensions of *S. mutans* and *P. gingivalis* (1.5 × 10^8^ CFU/mL) were prepared with 5% (*w*/*v*) sucrose. Sterilized disc-shaped specimens (*n* = 3; 10 mm × 2 mm) were placed into 24-well plates, each containing 1 mL of bacterial suspension and incubated at 37 °C for 48 h to allow biofilm formation and maturation [27]. The *S. mutans* was incubated aerobically, while *P. gingivalis* was incubated anaerobically. A positive control for growth control (broth + bacteria) and a negative control for sterile control (broth only) were included.

Following incubation, the samples were rinsed three times with PBS to remove non-adherent bacteria and air-dried at room temperature for several minutes. Then, the samples were stained with 0.1% (*w*/*v*) crystal violet for 20 min. Afterwards, the excess stain was gently washed three times with PBS. For quantitative assessment, the biofilm was dissolved by immersing each sample in 1 mL of 95% ethanol with gentle agitation for 45 min. The optical density (OD630) of the solubilized biofilm was measured using a microplate reader (ELX-800, BioTek, USA) to determine biofilm mass. The cut-off optical density (ODc) was calculated as the mean OD of the negative control plus three standard deviations and was used to classify the type of biofilm formation [27].

### 2.9. Optical Transparency Measurement

The transparency was measured using a UV–visible spectrophotometer (BK-D560, Biobase Co., Jinan, China) by placing each sample (*n* = 9; 9.5 mm × 50 mm × 0.5 mm) in the sample holder and recording the transmittance three consecutive times for each sample over the wavelength range of 350–750 nm. For comparative analysis, the transparency was quantified as the percentage area under the transmittance curve (AUC), calculated using the OriginPro software (OriginLab, OriginPro 2018, Northampton, MA, USA). The measured area was normalized to the theoretical maximum corresponding to 100% transmittance over the 350–750 nm wavelength range (400 nm interval). Transparency was calculated using the following equation [25]:

(1) Transparency(%)=(AUC÷[100×(750−350)]×100

### 2.10. Degree of Conversion (DC%)

Disc-shaped specimens (*n* = 9) with a diameter of 10 mm and thickness of 1 mm were analyzed for DC% using FTIR-ATR spectroscopy (IRSpirit-T, Shimadzu, Kyoto, Japan) equipped with QATR-S single-reflection ATR. The spectra of uncured resin and cured specimens were acquired over the spectral range of 4000–400 cm^−1^, with a resolution of 8 cm^−1^ and 32 scans per spectrum. The 810 cm^−1^ band was selected because it is well resolved and less affected by overlapping absorptions, allowing more reliable baseline determination. This band has also been reported to provide more reproducible measurements with lower variability for methacrylate conversion in closely related Tera Harz TC-85 resin [24]. Accordingly, the DC% was calculated from the reduction in the absorbance intensity of the 810 cm^−1^ band, corresponding to the out-of-plane bending (=CH_2_ wagging) vibration of the aliphatic acrylate C=C double bond after polymerization, according to the following equation [24]:

(2) $$DC(\%)=[(A_{810}uncured-A_{810}cured)÷A_{810}uncured]\times 100$$

### 2.11. Mechanical Properties

Tensile specimens (*n* = 9) were fabricated as dumbbell-shaped bars (length = 63.5 mm, width = 9.53 mm, thickness = 2 mm, and gauge width = 3.18 mm), according to ASTM D638-V [28]. Testing was conducted using a universal testing machine (Cussons technology Ltd., Manchester, UK). A constant crosshead speed of 1 mm/min was applied, with continuous recording of load and deformation until fracture. The three-point bending test was used to assess the flexural properties of a rectangular bar specimen (*n* = 9; 64 mm × 10 mm × 3.3 mm), according to ISO 20795-2 [29], on the same machine at a crosshead speed of 5 mm/min, with deflection forces continuously recorded throughout the test.

### 2.12. Statistical Analysis

Statistical analysis was performed using the SPSS software (IBM SPSS v25, Armonk, NY, USA). Data normality was assessed using the Shapiro–Wilk test (*p* > 0.05). The CFU counts were transformed to log_10_. Differences among groups in bacterial adherence assay (log_10_ CFU/mL), biofilm assay, transparency, DC (%), and mechanical tests were analyzed using one-way ANOVA followed by Tukey’s HSD post hoc test (*p* < 0.05).

## 3. Results

### 3.1. Physicochemical Characterization of Chitosan Nanoparticles Before and After Silanization

#### 3.1.1. FTIR

**Figure 1:**
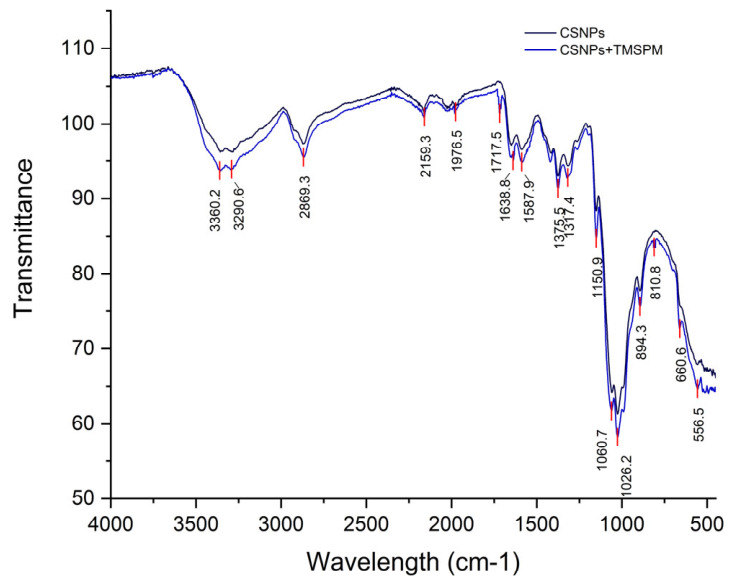
FTIR spectra of CSNPs before and after silanization.

**Figure 2:**
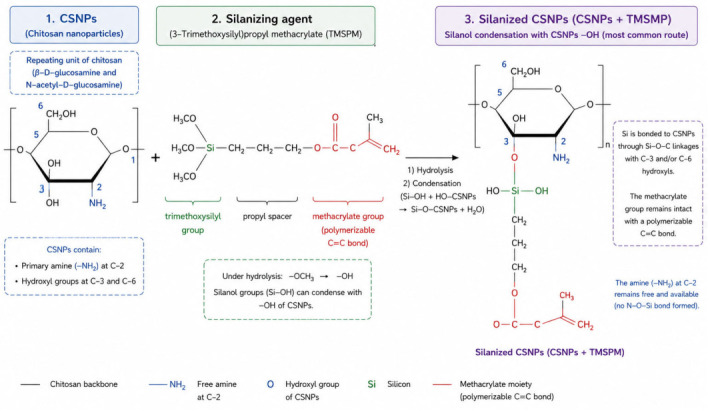
Schematic illustration of silanization of CSNPs with TMSPM.

The CSNPs showed distinguishing bands at 3360–3291 cm^−1^, which correspond to hydroxyl (O-H) and amino (N-H) stretching vibration, 2869 cm^−1^, corresponding to aliphatic carbon–hydrogen (C-H) stretching vibration, 1638 cm^−1^, representing the amide I vibration related to C=O stretching, and 1588 cm^−1^, attributed to amide II vibration resulting from N-H bending, along with polysaccharide backbone vibrations at 1151 and 1061–1026 cm^−1^ and β-glycosidic linkage at 894 cm^−1^ [30]. Following silanization, effective grafting was confirmed by a new peak at 1717 cm^−1^, which corresponds to the methacrylate ester carbonyl (C=O stretching) of TMSPM. The 1638 cm^−1^ region showed a slight spectral change due to overlap between the chitosan amide I band and the methacrylate C=C stretching vibration, while the characteristic =CH_2_ out-of-plane wagging band at 810 cm^−1^ further confirmed the incorporation of the methacrylate group. Furthermore, new low-frequency bands (≈660–556 cm^−1^), attributed to Si–O–Si vibrations, confirmed the development of the siloxane network, while enhanced absorption in the 1061–1026 cm^−1^ region, indicating covalent bonding between the silane and chitosan surface (Si−O−C linkages) [31,32]. The preservation of the main CSNPs peaks shows that surface modification did not degrade the polymer backbone (Figure 1 and Figure 2).

#### 3.1.2. UV–Visible Spectroscopy

**Figure 3:**
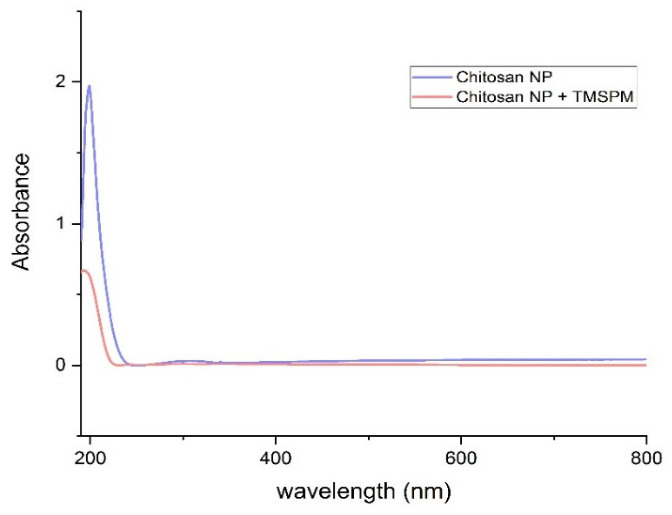
UV–visible spectroscopy of CSNPs before and after silanization.

A prominent absorption band in the wavelength range of ~200–230 nm was observed for the CSNPs, which is indicative of the transition of polysaccharide electronics and its functional groups (OH and −NH_2_) [33,34]. Following the TMSPM silanization, the overall spectral profile did not change, although the absorbance strength reduced, which showed less aggregation-induced scattering (Figure 3).

#### 3.1.3. TEM and EDS

**Figure 4:**
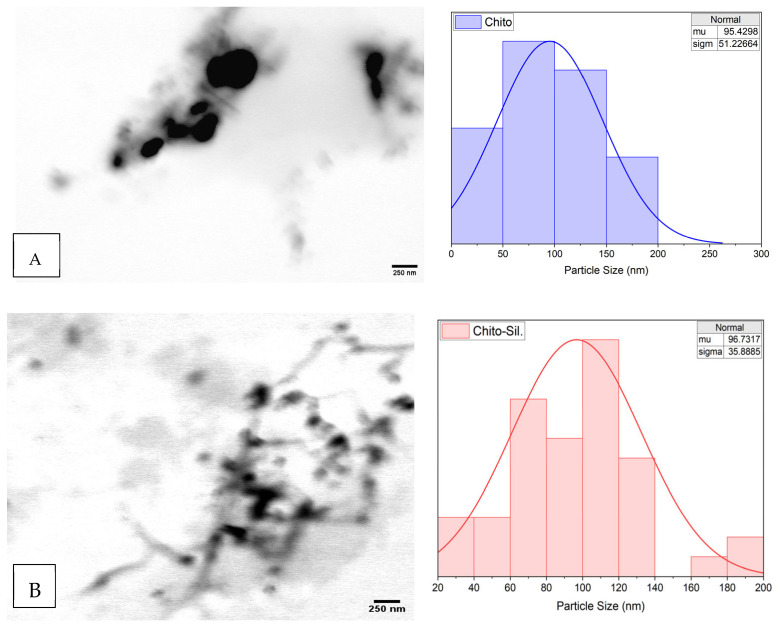
TEM: (**A**) CSNPs; (**B**) silanized CSNPs.

**Figure 5:**
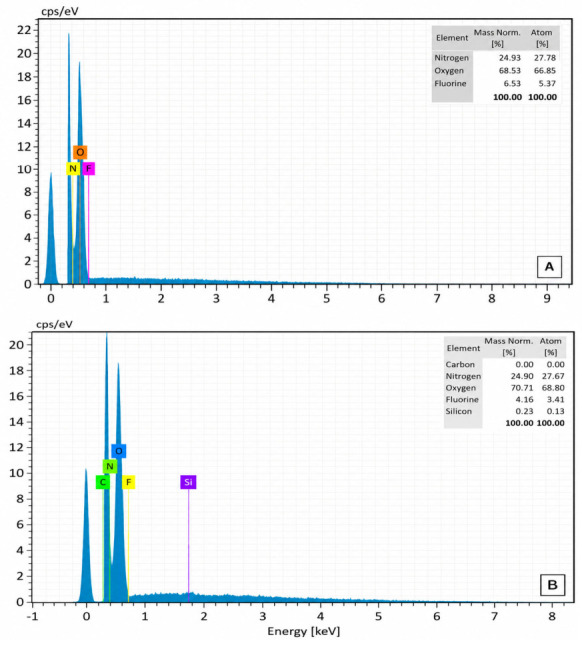
EDS: (**A**) CSNPs; (**B**) silanized CSNPs.

The TEM analysis (Figure 4A,B) showed that the CSNPs and silanized CSNPs had a spherical morphology, with mean particle sizes of 95.43 nm (σ = 51.23 nm) and 96.73 nm (σ = 35.89 nm), respectively. However, the narrower size distribution after silanization suggests improved uniformity. The silanized nanoparticles revealed diffuse borders and enhanced interparticle connection, which are in line with silane surface alteration. The silanized CSNPs included silicon (0.23 wt%), according to EDS analysis, whereas the unmodified nanoparticle showed no silicon signal (Figure 5A,B).

#### 3.1.4. XRD

**Figure 6:**
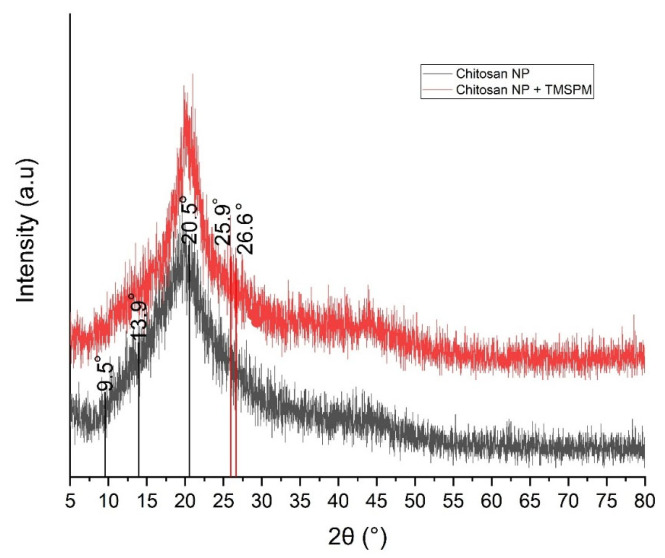
XRD of CSNPs before and after silanization.

The XRD pattern of the CSNPs showed distinctive reflections at 2θ = 9.54°, 13.93°, and 20.53°, which correspond to hydrated crystalline domains and chitosan interchain packing. A semi-crystalline structure typical of nanoscale chitosan and limited long-range order were indicated by the considerable widening of the prominent peak at 20.53° (d = 4.33 Å) [33]. However, the silanized CSNPs showed a prominent broad reflection at 2θ = 20.15° (d ≈ 4.41 Å), with suppression of low-angle crystalline peaks, implying the hydrogen-bonding domains after silanization were disrupted. Furthermore, the new reflections at 2θ ≈ 25.90° and 26.61° are attributed to siloxane (Si−O−Si) structural ordering, which confirms successful incorporation of the silane coupling agent and that the silanization resulted in an amorphous-dominated structure (Figure 6).

#### 3.1.5. DLS and Zeta Potential

**Figure 7:**
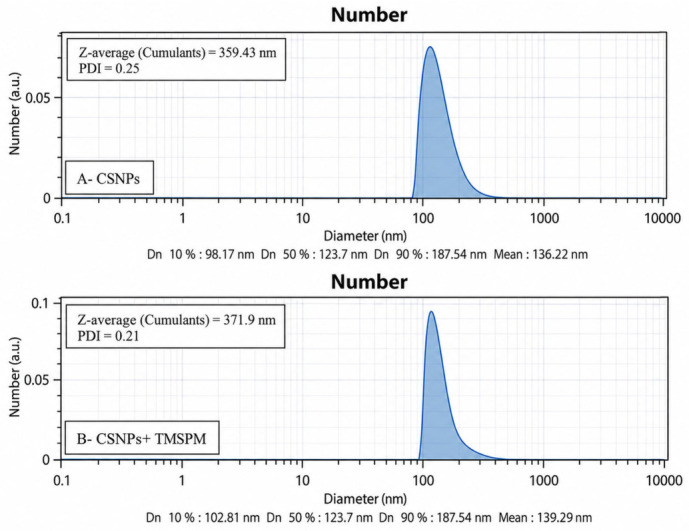
DLS: (**A**)—CSNPs; (**B**)—silanized CSNPs.

The CSNPs had a Z-average of 359.43 nm (PDI = 0.25), indicating moderate polydispersity. However, the silanized CSNPs’ Z-average increased to 371.9 nm (PDI = 0.21), showing similar dispersion behavior. The actual nanoparticle population is represented by the number-weighted size distribution. The CSNPs and silanized CSNPs had mean diameters of 136.32 nm and 139.39, respectively, demonstrating that the surface modification had minimal effect on the core sizes of the nanoparticles (Figure 7).

**Figure 8:**
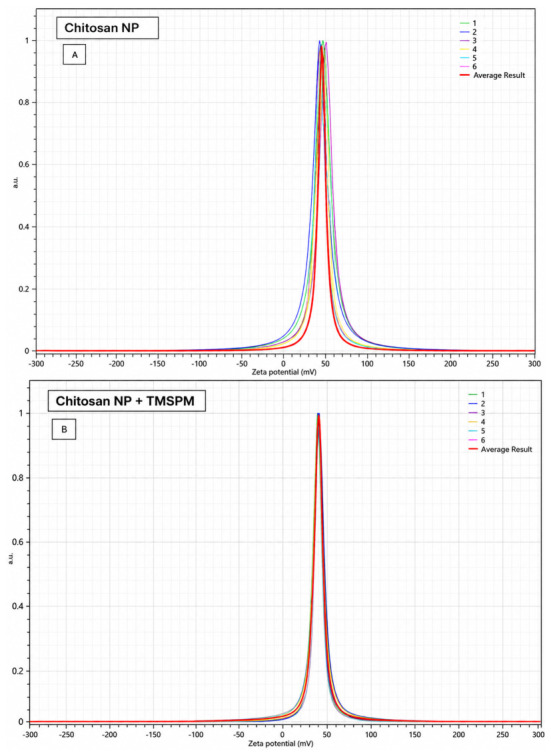
Zeta potential: (**A**) CSNPs; (**B**) silanized CSNPs.

Zeta-potential analysis revealed the CSNPs had a high positive surface energy (+45.38 ± 2.47 mV), suggesting excellent colloidal stability. Moreover, the silanized CSNPs exhibited a slightly reduced zeta potential (+38.59 ± 0.74 mV), although it stayed in a highly stable range (>+30 mV). After silanization, the reduced standard deviation indicated enhanced surface homogeneity and good grafting without substantial neutralization of protonated amine groups. This verifies that TMSPM modification preserved electrostatic stability without changing the positive surface charge of the CSNPs (Figure 8).

### 3.2. Antibacterial Evaluation

#### 3.2.1. MIC and MBC

The MIC for both the CSNPs and silanized CSNPs against both bacterial species was 0.125 mg/mL. The MBC for *S. mutans* was 0.25 mg/mL, and for *P. gingivalis*, it was 0.5 mg/mL. The MBC/MIC (≤4) ratios indicate bactericidal properties.

#### 3.2.2. Bacterial Adherence Assay

**Figure 9:**
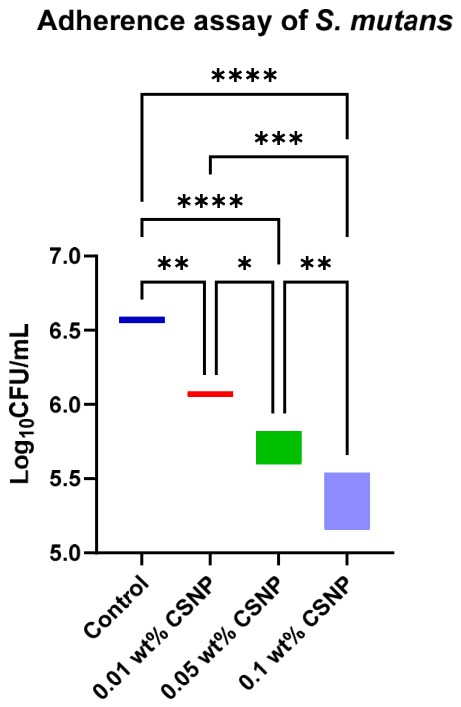
Adherence assay of *S. mutans*. Significance levels: * *p* < 0.05; ** *p* < 0.01; *** *p* < 0.001; **** *p* < 0.0001.

For both *S. mutans* and *P. gingivalis,* a statistically significant difference was observed among the groups (*p* < 0.001). The nano-modified groups significantly reduced *S. mutans* counts compared with the control group (*p* < 0.002). The incorporation of 0.01 wt%, 0.05 wt%, and 0.1 wt% nanoparticles resulted in 0.51, 0.83, and 1.23 log_10_ CFU/mL reductions in *S. mutans* relative to the control group, respectively. This reduction corresponded to approximately three-, seven-, and 17-fold decreases in CFU counts compared with the control group (Table 1; Figure 9).

**Figure 10:**
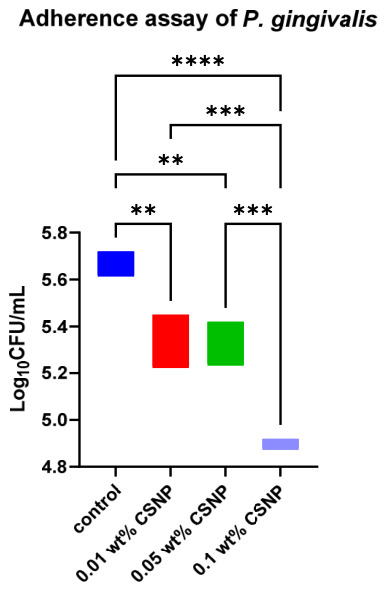
Adherence assay of *P. gingivalis.* Significance levels: ** *p* < 0.01; *** *p* < 0.001; **** *p* < 0.0001.

The post hoc test showed that the nano-modified groups had significantly reduced *P. gingivalis* counts compared with the control group (*p* < 0.005). The incorporation of 0.01 wt%, 0.05 wt%, and 0.1 wt% nanoparticles resulted in 0.31, 0.35, and 0.77 log_10_ CFU/mL reductions in *P. gingivalis* relative to the control group and corresponded to approximately two-, two-, and six-fold decreases in CFU counts in comparison with the control group. Furthermore, no significant difference was found between the 0.01% and 0.05% groups (*p* > 0.05). These findings demonstrate concentration-dependent antibacterial effects for both species (Table 1; Figure 10).

#### 3.2.3. Biofilm Assay

Biofilm quantification (OD_630_) showed a noticeable reduction following the incorporation of silanized CSNPs for *S. mutans* and *P. gingivalis* (Table 2). For *S. mutans,* the control group exhibited strong biofilm formation (0.38 ± 0.02), exceeding 4xODc (ODc = 0.069). In contrast, for the groups modified with 0.01 wt%, 0.05 wt%, and 0.1 wt% nanoparticles, the OD values reduced to 0.17 ± 0.04, 0.16 ± 0.03, and 0.13 ± 0.0, respectively. Thus, the 0.01 wt% and 0.05 wt% nanoparticle groups were classified as moderate biofilm producers, while the 0.1 wt% nanoparticle group was classified as a weak biofilm producer. Similarly, for *P. gingivalis*, the control group showed strong biofilm formation (0.13 ± 0.003), exceeding 4xODc (ODc = 0.030), whereas all groups modified with nanoparticles (0.01 wt%, 0.05 wt%, and 0.1 wt%) showed substantially lower OD values (0.063 ± 0.005, 0.056 ± 0.011, and 0.056 ± 0.005, respectively), corresponding to weak biofilm formation (≤ 2 × ODc).

**Figure 11:**
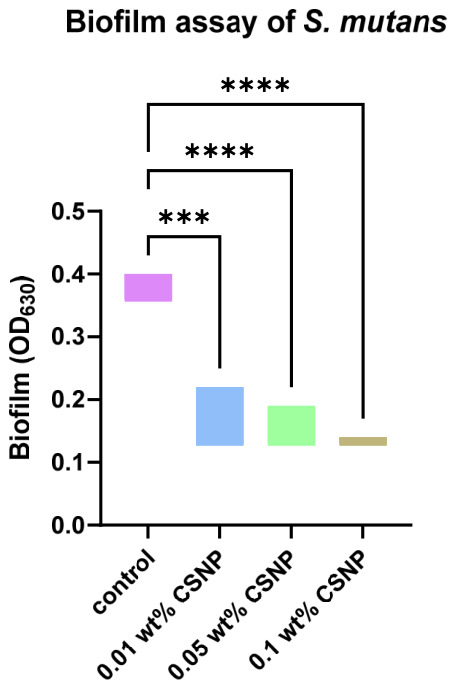
Biofilm assay of *S. mutans*. Significance levels: *** *p* < 0.001; **** *p* < 0.0001.

**Figure 12:**
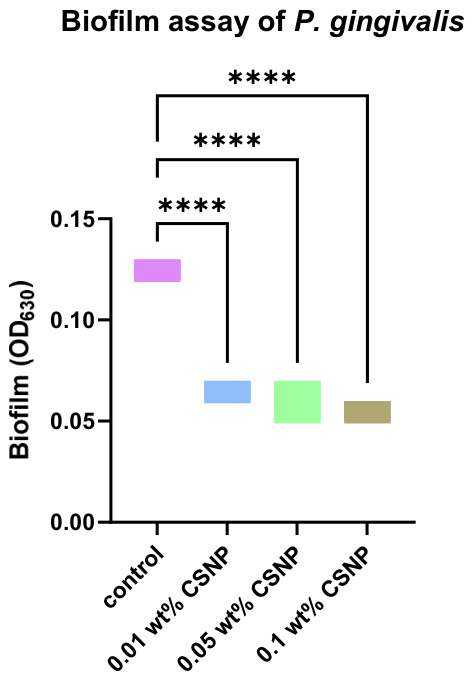
Biofilm assay of *P. gingivalis.* Significance levels: **** *p* < 0.0001.

Significant differences were observed among groups (*p* < 0.001) for both *S. mutans* (Figure 11) and *P. gingivalis* (Figure 12). The post hoc test confirmed that all nano-modified groups showed significantly lower biofilm formation in comparison with the control group (*p* < 0.001); however, no significant differences were observed between different concentrations (*p* > 0.05), demonstrating a non-dose-dependent effect within the tested range.

### 3.3. Optical Transparency Measurement

**Figure 13:**
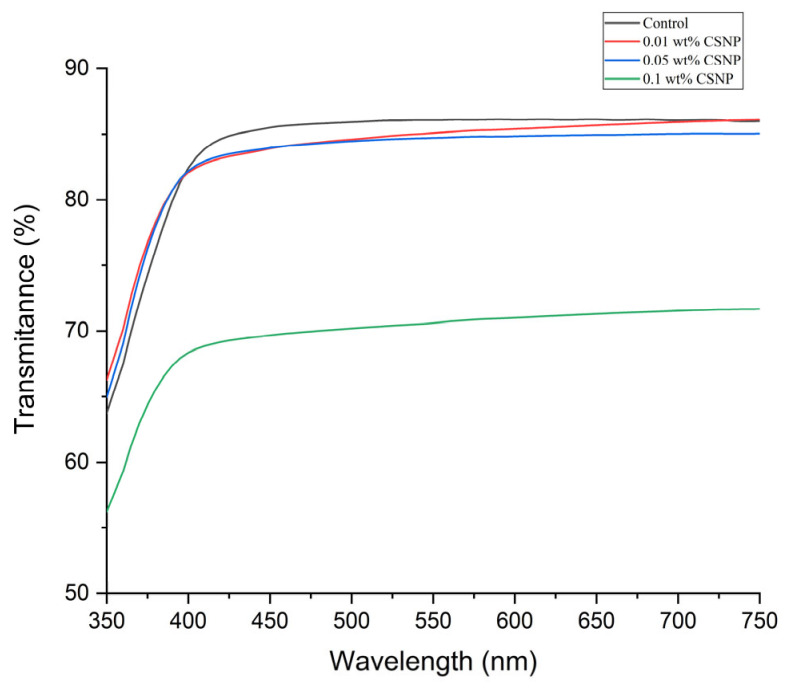
Optical transparency.

The control group showed the highest transmittance across the visible wavelength range (350–750 nm). The incorporation of 0.01 wt% and 0.05 wt% CSNPs reduced transmittance minimally, as the curves remained very similar to that of the control group. In contrast, the 0.1 wt% CSNP group showed a clear reduction in transmittance across the entire wavelength range, revealing reduced transparency (Figure 13).

These observations were confirmed by the quantitative analysis of the percentage area under the transmittance curve (Table 3). The transparency values were 84.29 ± 1.80 for the control group, 83.74 ± 2.45 for the 0.01 wt% CSNP group, 83.37 ± 1.19 for the 0.05 wt% CSNP group, and 69.76 ± 0.98 for the 0.1 wt% CSNP group. A significant difference was detected among the groups (*p* < 0.001). The post hoc test showed that the 0.1 wt% CSNP group exhibited significantly lower transparency than the other groups (*p* < 0.001).

### 3.4. Degree of Conversion

**Figure 14:**
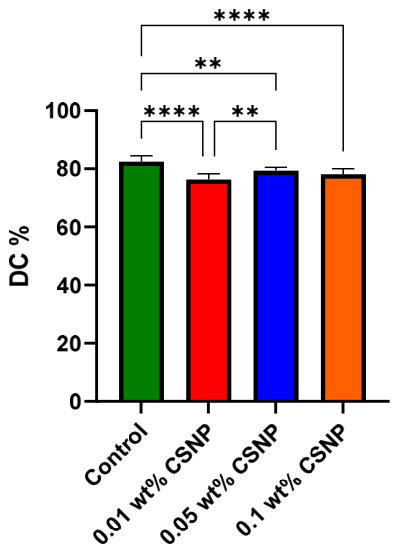
Degree of conversion. Significance levels: ** *p* < 0.01; **** *p* < 0.0001.

The degree of conversion was significantly affected by the nanoparticle incorporation. The control group exhibited the highest mean DC% (82.46 ± 1.96), followed by the 0.05 wt% CSNP group (79.40 ± 1.15), the 0.1 wt% CSNP group (78.15 ± 1.87), and the 0.01 wt% CSNP group (76.28 ± 2.02). The pairwise comparison showed that the control group had a significantly higher DC% than the nano-modified groups (*p* < 0.001). The 0.05 wt% CSNP group showed a significantly higher DC% than the 0.01 wt% group (*p* = 0.004). However, no significant differences were observed between the 0.1 wt% CSNP with 0.01 wt% and 0.05 wt% CSNP groups (Figure 14).

**Figure 15:**
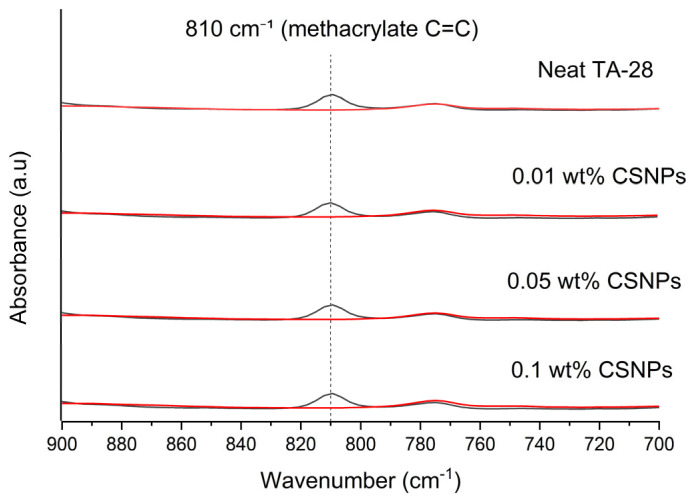
FTIR spectra before and after curing. The red lines show after curing.

Figure 15 represents FTIR spectra in the 900–700 cm^−1^ region, revealing a significant decrease in the absorption band at 810 cm^−1^, corresponding to the methacrylate vinyl group associated with a carbon–carbon double bond (C=C). The reduction in the peak intensity after curing confirms the consumption of reactive methacrylate double bonds and successful polymer network formation. The quantitative evaluation of the 810 cm^−1^ band showed that CSNP incorporation affected the polymerization, which was dependent on their concentration, with the 0.05 wt% CSNP group exhibiting the highest degree of conversion among the nano-modified groups.

### 3.5. Mechanical Properties

**Figure 16:**
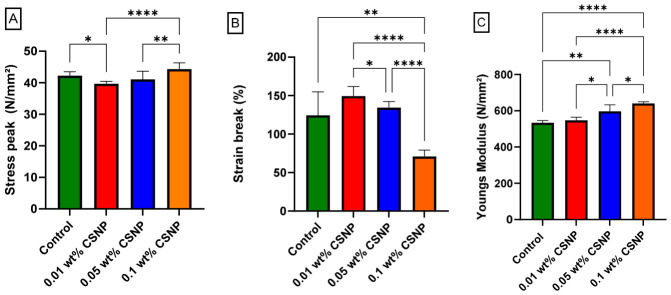
Tensile properties: (**A**) stress peak (N/mm^2^); (**B**) strain at break (%); and (**C**) Young’s modulus (N/mm^2^). Significance levels: * *p* < 0.05; ** *p* < 0.01; **** *p* < 0.0001.

The incorporation of CSNPs significantly affected the tensile behavior of the resin in a concentration-dependent manner. The stress peak differed significantly among the groups (*p* < 0.001). The 0.01 wt% group showed a decrease compared with the control group, in contrast with the 0.1 wt% group, exhibiting the highest values. The 0.05 wt% group showed no significant difference from the control group (Figure 16A). The strain at break also showed significant differences among the groups (*p* < 0.001). The 0.01 wt% group demonstrated the highest elongation, whereas the 0.1 wt% group showed a marked and significant reduction compared with all other groups, indicating reduced ductility (Figure 16B). Young’s modulus increased significantly with increasing nanoparticle concentration (*p* < 0.001). The 0.01 wt% group did not significantly differ from the control group, while both 0.05 and 0.1 wt% groups showed significantly higher stiffness (Figure 16C). Overall, as the nanoparticle concentration increased, the stiffness and tensile strength improved, but reduced the elongation at break.

**Figure 17:**
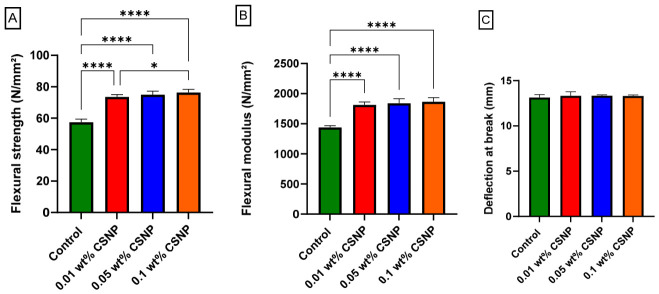
Flexural properties: (**A**) flexural strength (N/mm^2^); (**B**) flexural modulus (N/mm^2^); (**C**) and deflection at break (mm). Significance levels: * *p* < 0.05; **** *p* < 0.0001.

Similarly, the flexural properties were significantly affected by the incorporation of CSNPs (*p* < 0.001). All nano-modified groups showed higher flexural strength than the control group, with strength increasing progressively as the nanoparticle concentration increased (Figure 17A). The flexural modulus also increased significantly after nanoparticle incorporation (*p* < 0.001), indicating greater stiffness and resistance to bending deformation. However, no significant differences were detected among the nano-modified groups (Figure 17B). In contrast, deflection at break was not significantly affected (*p* = 0.435), suggesting that adding the nanoparticle did not compromise the material’s flexibility during bending (Figure 17C).

## 4. Discussion

This study explored whether the incorporation of silanized CSNPs with TMSPM into direct-printed clear aligner resin (TA-28) could reduce bacterial adhesion and biofilm formation while maintaining the esthetic requirements essential for clinical use. The findings did not reveal a consistent improvement across all evaluated properties. Instead, they demonstrated a concentration-dependent optimization window, whereby moderate reductions in bacterial adhesion and biofilm formation were accompanied by changes in transparency and ductility. As a result, concentrations that improved antibacterial performance did not necessarily preserve the optical or mechanical properties.

The TMSPM was used as a silane coupling agent to improve the interaction between chitosan nanoparticles and the methacrylate-based resin matrix, as the hydrolyzed silane groups would covalently bond to the hydroxyl group on the chitosan surface, and the methacrylate functional group would remain available to co-polymerize with the resin matrix, as this would enhance the interfacial adhesion and improve the dispersion of nanoparticles, eventually optimizing the integrity of the composite system [35]. The findings of this study confirm the efficacy of silanization, a well-established method in dental materials science for improving filler–matrix compatibility. The characteristics of the FTIR signals, together with silicon detected by EDS, confirm that the surface modification was successful. Following the silanization, the chitosan nanoparticles’ positive surface charge was retained, although slightly reduced. This is necessary because the antibacterial activity of chitosan is driven by its cationic nature, which permits interaction with negatively charged bacterial cell membranes and results in membrane breakdown and cell death [36].

The comparable MIC values imply that silanization does not change the inherent antibacterial properties of chitosan nanoparticles. Moreover, rather than growth inhibition, the bactericidal action, as indicated by MBC/MIC ratios ≤ 4, shows the direct breakdown of the bacterial membrane. It is well known that chitosan electrostatically interacts with negatively charged bacterial cell walls, leading to increased membrane permeability, leakage of intracellular contents and, eventually, cell death [37]. However, a higher MBC for *P. gingivalis* was observed, maybe due to its Gram-negative structure, as the outer membrane, enriched in lipopolysaccharide, acts as a protective barrier and minimizes the bactericidal action. In contrast, the Gram-positive *S. mutans* lacks this outer membrane, which is composed of peptidoglycan, making it more vulnerable to the membrane-targeting mechanism [38]. This difference in structure supports the consistently stronger antibacterial response for *S. mutans* throughout all assays.

Previous studies have demonstrated that incorporating nanoparticles can enhance clear aligners’ resistance to biofilm formation [1,3,39]. In the present study, Graphy TA-28 3D-printed resin was chosen as the base material for functionalization because of its advantageous physical characteristics. Its shape-memory behavior at intra-oral temperature improves the aligner adaptation, minimizes the force decay over time, and allows more efficient tooth movement in each treatment step [9]. Furthermore, previous studies have shown that the mechanical stability is maintained for one week of intraoral use [40]. Despite these promising features, the material does not possess intrinsic antimicrobial activity, which is a key limitation and justifies the need for further functional modification.

Following the incorporation of silanized chitosan nanoparticles into the resin, the results show that antibacterial activity was retained, as a frequent concern with incorporating silanized nanoparticles within the resin is that it could limit their release and weaken their antibacterial effect. The results show that release may not be the dominant mechanism, but rather seems contact-driven, as positively charged chitosan groups at the surface of the resin interact directly with bacterial membranes and hinder colonization. Furthermore, immobilizing nanoparticles within the resin help to reduce leaching, which is valuable for intraoral safety. Overall, modification with TMSPM led to a biomaterial that has antibacterial performance and stability [41,42].

The bacterial adherence assay revealed a statistically significant reduction in viable adherent bacteria to the nano-modified groups compared with the control group for both species (*p* < 0.05). However, the magnitude of the reduction was limited, ranging from 0.51 to 1.23 log_10_ CFU/mL for *S. mutans* and from 0.31 to 0.77 log_10_ CFU/mL for *P. gingivalis* across the nano-modified groups. The highest reduction was observed at 0.1 wt%; however, the overall reduction remained approximately one order of magnitude relative to the control group. These findings show that the main effect of silanized CSNPs is to inhibit initial bacterial adhesion rather than conferring a bactericidal surface [43]. The concentration-dependent reduction in *S. mutans* adherence suggests that increasing the nanoparticle concentration may have increased the availability of antibacterial sites on the material surface, which increases the likelihood of bacterial interaction and membrane disruption. Meanwhile, *P. gingivalis* demonstrated a less pronounced concentration response and did not differ significantly between the lower concentrations (0.01 wt% and 0.05 wt%). This may be due to the resistance of its outer membrane and lipopolysaccharide layer and its fimbriae (FimA and Mfa1) adhesion system [44], which might be less susceptible to surface charge changes and limit contact-mediated antibacterial interactions.

Attachment of bacteria is the first step in the process of biofilm formation, which is followed by proliferation, synthesis of extracellular polymeric substances (EPSs), and maturation [45]. Therefore, inhibition of early attachment will have a direct effect on biofilm formation. This was observed in the present study, as a significant reduction in bacterial adherence corresponded to a marked reduction in biofilm mass for both *S. mutans* and *P. gingivalis* after 48 h. The lack of difference between the concentrations to reduce the biofilm mass suggests a threshold effect, where even a low nanoparticle level (0.01 wt%) can sufficiently reduce the initial bacterial attachment, limiting further biofilm formation. Although higher concentrations improved early antibacterial activity (Log_CFU reduction), this was not seen in the biofilm assay. Moreover, the crystal violet assay quantifies total biomass (cell and extracellular matrix), which may mask the concentration-dependent response as it does not distinguish between viable and non-viable bacteria [46].

Biofilm type can be classified as weak, moderate, or strong based on cut-off (ODc) values using optical density (OD)-based classification. Strong biofilm formation (≥4ODc) is related to higher pathogenic ability and more resistance to interventions [16]. The shift observed in the present study from a strong biofilm type in the control group to a moderate or weak biofilm type in the nano-modified groups is clinically meaningful as it represents a substantial reduction in microbial burden.

Compared with previous studies, the antibacterial properties were lower than those of clear aligners coated with TiO_2_‚ ZnO‚ selenium‚ gold‚ and quaternary ammonium coatings. They demonstrated greater inhibition of bacterial growth and biofilm formation‚ though often needed to be activated‚ had unwanted effects on optical properties‚ and/or increased the surface roughness [1,27,47,48,49]. The findings of lower antibacterial properties in this study may be explained by the bulk incorporation of CSNPs into the resin‚ resulting in only a small quantity of immobilized CSNPs being present on the top surface of the resin‚ which is in contact with the bacteria. However‚ the silanized CSNPs are covalently bonded to the methacrylate polymer chain and would be retained better whilst minimizing coating degradation or particle leaching during clinical use. Although bulk incorporation may not exhibit the same antibacterial effects as surface functionalization‚ the possibility of high stability and persistency of the material may be helpful.

While the incorporation of CSNPs significantly reduced the bacterial adhesion and biofilm formation for both species, these benefits must be considered alongside the changes in transparency. Low concentrations of nanoparticles (0.01–0.05 wt%) retained the optical transparency comparable to the control group, suggesting sufficient nanoparticle dispersion within the resin and minimal light scattering. In contrast, the 0.1 wt% nanoparticle concentration significantly reduced the optical transparency in comparison with the control group, indicating the limit where optical properties begin to decline, likely due to increased light scattering with higher nanoparticle loading. Although the 0.1 wt% nanoparticle concentration resulted in the greatest reduction in bacterial adherence, it also caused a significant loss of transparency, while providing no additional reduction in mature biofilm compared with lower nanoparticle concentrations. This reveals that the higher nanoparticle concentration provided limited antibacterial benefit while compromising the optical properties. Hence, within the nano-modified groups, 0.05 wt% represents a favorable balance by preserving transparency while achieving greater antibacterial activity than 0.01 wt% without the marked reduction in optical transparency associated with 0.1 wt%. Similar findings have been reported for TiO_2_-coated clear aligners, where lower nanoparticle concentrations preserved the optical transparency [1].

Alongside the optical properties, the polymerization behavior of the resin was evaluated using the degree of conversion. All nano-modified groups had lower DC values than the control group, although the values remained within the clinically acceptable range. Interestingly, the relationship between nanoparticle concentration and DC was not linear. Among the nanomodified groups, the 0.05 wt% CSNPs had the highest DC, while the 0.01 wt% and 0.1 wt% CSNPs had lower DC values. This suggests that polymerization was influenced not only by nanoparticle concentration but also by the interaction between the nanoparticles and the resin matrix. Factors such as nanoparticle dispersion, the restriction of monomer mobility, and light transmission during photopolymerization may have contributed to this behavior; however, because the nanoparticle dispersion within the cured resin was not evaluated, these mechanisms remain speculative. Notably, the concentration that exhibited the highest antibacterial activity (0.1 wt%) did not correspond to the highest DC, further revealing that enhancing antibacterial performance does not necessarily enhance polymerization behavior. Despite this reduction in DC, all groups maintained a DC above 75%, indicating adequate polymerization for clinical applications. These values are comparable to the reported DC of TC-85A resin (≈83%). Maintaining the DC within this range is important because higher degrees of conversion are associated with improved mechanical properties, chemical stability, and biocompatibility [50].

The tensile properties demonstrated a clear stiffness–ductility trade-off rather than uniform mechanical improvement following the incorporation of silanized CSNPs. The improvement in the tensile strength at 0.1 wt% CSNPs shows better load transfer between the polymer matrix and the nanoparticles, which can be linked to enhanced interfacial bonding achieved by TMSPM silanization. Covalent bonds are formed between the hydrophilic chitosan nanoparticle surface and the resin matrix, which increases the resistance to fracture by assisting stress distribution under tensile loading [51,52]. Conversely, the decrease in tensile strength at 0.01 wt% CSNPs likely reflects limited interaction between the nanoparticles and the polymer matrix. As the filler content is below the critical threshold needed to form an effective reinforcing network, the nanoparticles may behave more like concentration sites rather than reinforcing elements, thereby limiting effective stress transfer within the resin. The nanoparticle concentration of 0.05 wt% CSNPs appears to represent a transition zone, where better dispersion and partial network formation begin to enhance mechanical stability without compromising flexibility.

The stiffening effect of CSNP incorporation is further confirmed by the gradual increase in Young’s modulus with an increasing nanoparticle concentration. This behavior is related to the restricted mobility of polymer chains due to strong filler–matrix interactions, resulting in a stiffer composite structure. This behavior conforms with the mechanism described by Molinari et al. [53], where mechanical reinforcement improves when nanoparticles create effective networks of polymer–particle interactions, transmitting applied stresses across the matrix. However, the increase in stiffness at 0.1 wt% was associated with a marked drop in strain at break, indicating reduced ductility. Thus, the concentration that achieved the highest tensile strength also produced the most brittle material; there is an inherent compromise between mechanical reinforcement and ductility. As clear aligners are repeatedly inserted, removed and subjected to continuous deformation, preserving sufficient ductility is as important as improving strength. Therefore, although a higher nanoparticle concentration enhanced stiffness and tensile strength, these aspects should be considered alongside the accompanying loss of ductility when selecting an appropriate nanoparticle concentration.

In contrast with the tensile properties, the flexural properties showed a favorable response following CSNP incorporation. All nano-modified groups exhibited significantly higher flexural strength and flexural modulus than the control group, though no significant differences were observed among the nano-modified groups. This suggests that even the lowest concentration (0.01 wt%) was sufficient to establish an effective reinforcing interphase between the CSNPs and the polymer matrix, thereby improving stress transfer under bending loads [54]. Further increases in the nanoparticle concentration did not result in additional reinforcement and suggested that the flexural properties had reached a plateau within the investigated concentration range. Meanwhile, deflection at break remained unchanged among the groups, showing the silanized CSNPs’ loading enhanced stiffness and bending resistance without compromising flexibility. This balance is advantageous for clear aligners, which require sufficient rigidity for force delivery while maintaining flexibility for insertion, removal, and patient comfort.

Overall, the current findings show that silanized CSNP incorporation does not uniformly improve all material properties and instead produces concentration-dependent trade-offs. Antibacterial activity showed a concentration-dependent response, with increasing CSNP concentration producing greater reduction in bacterial adherence, whereas biofilm inhibition plateaued at 0.01 wt%, with no significant differences among the nano-modified groups. The highest nanoparticle concentration (0.1 wt%) exhibited the highest antibacterial effect; it also compromised the optical transparency and ductility. In contrast, the 0.05 wt% CSNP group had the most balanced overall performance by preserving transparency, degree of conversion, and flexural and tensile strengths, and was superior to the 0.01 wt% CSNP group in antibacterial activity without the optical and ductility drawbacks observed in the 0.1 wt% CSNP group. Therefore, the 0.05 wt% CSNP group appears to be the concentration providing the most favorable balance between antibacterial activity and maintaining the physical and mechanical properties evaluated in the present in vitro study. Although the antibacterial effect remained below the level generally considered bactericidal, the absence of additional biofilm inhibition at higher CSNP concentrations suggests that simply increasing nanoparticle concentration is unlikely to provide further antibacterial benefit. This study demonstrates that silanized CSNPs can be successfully incorporated into a shape-memory, directly 3D-printed clear aligner resin (TA-28) and identifies a concentration range that balances antibacterial activity with preservation of the optical and mechanical properties required for clinical application.

This study has several limitations. First, the antibacterial assays were performed with a relatively small sample size, consistent with previous preliminary microbiological studies. Therefore, larger studies and in vivo studies are warranted. Second, the antibacterial activity was assessed over a short period, and the long-term antibacterial effect after prolonged intraoral exposure was not studied. Third, surface characteristics, such as surface roughness, wettability and the nanoparticle distribution on printed samples, were not evaluated. Finally, further studies are needed to investigate aging protocols, multispecies biofilm models and in vivo performance.

## 5. Conclusions

The incorporation of silanized CSNPs into directly 3D-printed clear aligner resin produced a concentration-dependent response rather than uniform improvement across all evaluated properties. While all nano-modified groups significantly reduced bacterial adhesion and biofilm formation, the 0.1 wt% CSNPs group achieved the greatest antibacterial activity, but this was accompanied by reduced transparency and ductility. In contrast, 0.05 wt% CSNPs demonstrated the most favorable balance between antibacterial efficiency, optical properties, polymerization behavior, and mechanical performance. These findings suggest that silanized CSNPs represent a potentially useful strategy for developing functional direct-printed clear aligner material with enhanced resistance to microbial colonization while preserving clinical properties. Furthermore, in vivo and long-term studies are required to confirm their clinical performance, durability, and biocompatibility.

## Figures and Tables

**Table 1:** Bacterial adherence assay (log_10_ CFU/mL).

Group	*n*	Mean ± SD (log_10_ CFU/mL) *S. mutans*	Mean ± SD (log_10_ CFU/mL) *P. gingivalis*
Control	3	6.58 ± 0.02 ^a^	5.67 ± 0.05 ^a^
0.01 wt%	3	6.07 ± 0.01 ^b^	5.35 ± 0.11 ^b^
0.05 wt%	3	5.75 ± 0.12 ^c^	5.32 ± 0.09 ^b^
0.1 wt%	3	5.34 ± 0.19 ^d^	4.90 ± 0.02 ^c^

^a–d^ One-way ANOVA (*p* < 0.001). Different superscript letters show significant differences between groups according to Tukey’s HSD post hoc test.

**Table 2:** Biofilm assay.

Group	*n*	Mean ± SD (OD_630_) *S. mutans*	Mean ± SD (OD_630_) *P. gingivalis*
Control	3	0.382 ± 0.023 ^a^	0.128 ± 0.003 ^a^
0.01 wt%	3	0.173 ± 0.045 ^b^	0.063 ± 0.006 ^b^
0.05 wt%	3	0.163 ± 0.031 ^b^	0.057 ± 0.012 ^b^
0.1 wt%	3	0.137 ± 0.006 ^b^	0.057 ± 0.006 ^b^

^a,b^ One-way ANOVA (*p* < 0.001). Different superscript letters indicate significant differences between groups according to Tukey’s HSD post hoc test.

**Table 3:** Transparency measurement.

Group	Transparency % (Mean ± SD)
Control	84.29 ± 1.80 ^a^
0.01 wt% CSNPs	83.74 ± 2.45 ^a^
0.05 wt% CSNPs	83.37 ± 1.19 ^a^
0.1 wt% CSNPs	69.76 ± 0.98 ^b^

^a,b^ One-way ANOVA (*p* < 0.001). Different superscript letters indicate significant differences between groups according to Tukey’s HSD post hoc test (*p* < 0.001).

## Data Availability

The original contributions presented in this study are included in this article; further inquiries can be directed to the corresponding author.

## Abbreviations

The following abbreviations are used in this manuscript:

ATR-FTIR	Attenuated total reflectance–Fourier-transform infrared spectroscopy
AUC	Area under the curve
CFU	Colony-forming unit
CSNPs	Chitosan nanoparticles
DC	Degree of conversion
DLS	Dynamic light scattering
EDS	Energy-dispersive spectroscopy
EPS	Extracellular polymeric substance
MIC	Minimum inhibitory concentration
MBC	Minimum bactericidal concentration
OD	Optical density
ODc	Cut-off optical density
PBS	Phosphate-buffered saline
PDI	Polydispersity index
*P. gingivalis*	*Porphyromonas gingivalis*
*S. mutans*	*Streptococcus mutans*
TA-28	Tera Harz TA-28 resin
TEM	Transmission electron microscopy
TMSPM	3-(trimethoxysilyl) propyl methacrylate
TPP	Sodium tripolyphosphate
UTM	Universal testing machine
UV-Vis	Ultraviolet–visible spectroscopy
WSLs	White spot lesions
XRD	X-ray diffraction
